# Circulating interleukin-6 and rheumatoid arthritis: A Mendelian randomization meta-analysis

**DOI:** 10.1097/MD.0000000000003855

**Published:** 2016-06-10

**Authors:** Bing Li, Yu Xiao, Dan Xing, Xin-long Ma, Jun Liu

**Affiliations:** Joint Department, Tianjin Hospital, Tianjin, China.

**Keywords:** IL-6 -174G/C polymorphisms, interleukin-6, mendelian randomization, meta-analysis, rheumatoid arthritis

## Abstract

Interleukin-6 (IL-6), as a pleiotropic cytokine, has been demonstrated to be closely associated with the pathogenisis of rheumatoid arthritis (RA). However, whether this association is causal or not remains unclear, because of the multifactorial role of IL-6 and related confounding factors. We aimed to evaluate the causal relevance between circulating IL-6 levels and the risk of RA through meta-analytical Mendelian randomization approach. *IL-6* gene -174G/C variant was selected as an instrument in this Mendelian randomization meta-analysis. Article identification and data collection were conducted in duplicate and independently by 2 authors. The STATA software was used for data analysis. In total, 15 and 5 articles on the association of the -174G/C variant with RA risk and circulating IL-6 level, respectively, were included. The overall analysis showed that C allelic and GC+CC genotype were significantly with 1.59-fold (95% CI: 1.19–2.14) and 1.63-fold (95% CI: 1.17–2.26) increased risk of developing RA, respectively. Asian populations showed stronger association with 4.55-fold (95% CI: 1.62–12.75), 1.84-fold (95% CI: 1.13–2.99), and 4.69-fold (95% CI: 1.68–13.14) increased RA risk in carriers of -174C allelic, CC, and GC+CC genotype, respectively. Carriers of GC+CC genotype showed significant reduction in the circulating IL-6 level compared with GG carriers (WMD = −0.77; 95% CI: −1.16 to −0.38; *P* = 0.000) in overall populations. Mendelian randomization presented 6% and 22% increased risk of RA with 0.1 pg/mL reduction of circulating IL-6 level in overall and Asian populations, respectively. This Mendelian randomization meta-analysis demonstrated that the long-term genetically reduced circulating IL-6 level might be causally related to a higher risk of RA, especially in Asian populations.

## 1 Introduction

Interleukin-6 (IL-6) is a pleiotropic cytokine with multiple functions in different pathophysiologic systems, including rheumatoid arthritis (RA).^[1,2]^ High concentrations of IL-6 in both the synovial fluid^[3,4]^ and serum^[5–7]^ of patients with RA suggested a major role of this cytokine in the pathogenesis of RA, which is further confirmed by the recent introduction of tocilizumab, a humanized monoclonal antibody targeting IL-6R.^[8]^ However, it remains unclear whether the relevance between circulating IL-6 and RA is causal, owing to the multifactorial role of IL-6 (both pro- and antiinflammatory)^[9]^ and to confounding or reverse causation, which is often inevitable in observational studies.

Mendelian randomization, a natural randomization process according to Mendel second law, is free of the confounding and reverse causation (typical of classical epidemiology) and has been developed to exploit the effect of long-term exposure differences on disease risk.^[10]^ It has become a promising alternative to well-designed randomized controlled trials for evaluating the causal relevance between phenotype and disease, when it is neither practical nor ethical to employ randomized human beings. Since its introduction in 2004,^[11]^ Mendelian randomization has been successfully applied in a wide variety of studies focusing on the causal relevance of genetic exposures with multifactorial disease, such as diabetes and the risk of developing coronary artery disease,^[12]^ vitamin D status, and the risk of hypertension.^[13]^

Although high concentrations of IL-6 were observed in RA patients, its levels are heterogeneous,^[6,7]^ which are considered to be largely genetically determined. The genomic sequence of IL-6 is highly polymorphic, and the promoter -174G/C (rs1800795) is one of the most frequently evaluated variants.^[14,15]^ Data regarding IL-6 levels across -174G/C variants are controversial; some studies have shown higher circulating IL-6 levels in GG carriers,^[16,17]^ whereas other studies have reported no difference among the genotypes or increased levels in CC carriers,^[18,19]^ suggesting the complicated role of genetics in determining circulating IL-6 level. Besides genes, other confounding factors, such as body mass index (BMI), age, drug regimens, and environmental factors, were also found to have an impact on circulating IL-6, which may bias the results regarding the association between the IL-6 level and RA risk. We thus performed this Mendelian randomized meta-analysis to exploit the causal relevance of circulating IL-6 with RA, using the *IL-6* gene *-174G/C* variant as an instrument.

## 2 Materials and methods

The present meta-analysis was carried out complying with the guidelines put forward by the Preferred Reporting Items for Systematic Reviews and Meta-analysis (PRISMA) statement.^[20]^ Ethical approval is waived, because no clinical and animal experiment was involved in this mendelian randomization meta-analysis.

### 2.1 Search strategy

Comprehensive research was performed to identify potentially relevant studies, using the following key terms: “interleukin 6,” “IL-6,” “rheumatoid arthritis,” “RA,” in combination with, “gene polymorphism,” “SNP,” “variation,” and “mutation.” The language was restricted to English and Chinese. Articles were sourced from PubMed, Embase, and China National Knowledge Infrastructure, from the earliest possible year to September, 2015. Bibliographies of the retrieved articles, reviews, and these were also checked for other relevant publications.

The titles and abstracts of all retrieved articles were independently read by 2 authors (XY and WL). In case of uncertain rejection, the full text and supplementary materials were reviewed to check whether the relevant data had been provided and if it was necessary to contact the authors to request additional information. Data were extracted from the most recent or complete article if more than one article from a study group was published. The eligibility was assessed in duplicate and independently by the same 2 authors. Any uncertainty over the eligibility was resolved by a discussion or further joint inspection of original articles.

### 2.2 Inclusion and exclusion criteria

Studies meeting the following criteria were included: data on associations between *IL-6* gene -174G/C variants and RA risk were provided; mean values and the corresponding standard deviation of circulating IL-6 were provided across *IL-6* gene G/C alleles or genotypes; and detailed genotype or allele counts of *IL-6* gene 174G/C variant were tractable between RA and healthy controls.

Conference abstracts, case reports, series, editorials, narrative and systematic reviews, and non-English and Chinese article were excluded. Articles that assessed the progression, severity, phenotypic modification, and response to treatment of RA in association with *IL-6* gene G/C variant, or that lacked patients or healthy controls, were not covered. Regarding the association between -174G/C variants and IL-6 circulating level, studies were also excluded if the average age of participants were more than 60 or less than 20, because of the remarkable change of serum IL-6 level in these populations.^[21–23]^

### 2.3 Data collection

Data were collected independently from each qualified article by the 2 authors (XY and WL), according to a predefined protocol, including the first author's last name, publication year, ethnicity, diagnostic criteria for RA, match condition between patients and controls, sample size, the allelic and genotype counts of *IL-6* gene 174G/C polymorphism between RA patients and controls, the mean (standard deviation) values of circulating IL-6 for each *IL-6* gene 174G/C allele and genotype, as well as some baseline characteristics of the study populations (when available), including age, gender, BMI, and medication history. The units of circulating IL-6 were uniformly standardized as pg/mL for consistency.

### 2.4 Statistical analysis

All the statistical analyses were performed with the use of STATA software (version 11.0 for Windows; Stata Corp, College Station, TX).

The association of *IL-6* gene G/C variant with RA risk was expressed as odds ratio (OR) and 95% confidence interval (95% CI). Weighted mean difference (WMD), as well as 95% CI, was calculated to compare the changes of circulating IL-6 level across genotype carriers. The departure of frequencies of IL-6 -174G/C variants from Hardy–Weinberg equilibrium (HWE) was tested by Chi-square test, and *P* < 0.05 was accepted as statistically significant.

Heterogeneity was quantified by the inconsistency index (*I*^2^). This statistic, which ranges from 0% to 100%, is defined as the percentage of the observed between-study variability that is due to heterogeneity rather than chance. In this meta-analysis, heterogeneity was defined “low” if the *I*^2^ values were <25%, “moderate” if the *I*^2^ values were between 25% and 75%, and “large” if the *I*^2^ values were >75%. The fixed effects model was selected to calculate the pooled ORs when the heterogeneity was low. Otherwise, the random effects model with the Dersimonian and Laird method was employed to bring individual effect size estimates together.

To evaluate the contribution of individual studies to pooled effect estimates, a sensitivity analysis was performed by sequentially omitting each study one at a time and computing differential estimates for remaining studies.

Publication bias was determined by the Begg funnel plot and Egger regression asymmetry test. An asymmetric plot suggests the possible presence of publication bias, which can be verified by Egger test that can detect funnel plot asymmetry by testing whether the intercept deviates significantly from 0 in regressing the standardized effects estimates against their precision. Statistical significance was considered when the *P* value of Egger test was less than 0.05.

Under the assumptions of Mendelian randomization, the potential causal relevance of circulating IL-6 level with RA risk can be reflected by the risk estimates, which is calculated by the ratio of the coefficient of the association between *IL-6* gene -174G/C variant and RA to that of the association between -174G/C variant and circulating IL-6.

## 3 Results

### 3.1 Eligible studies

**Figure 1:**
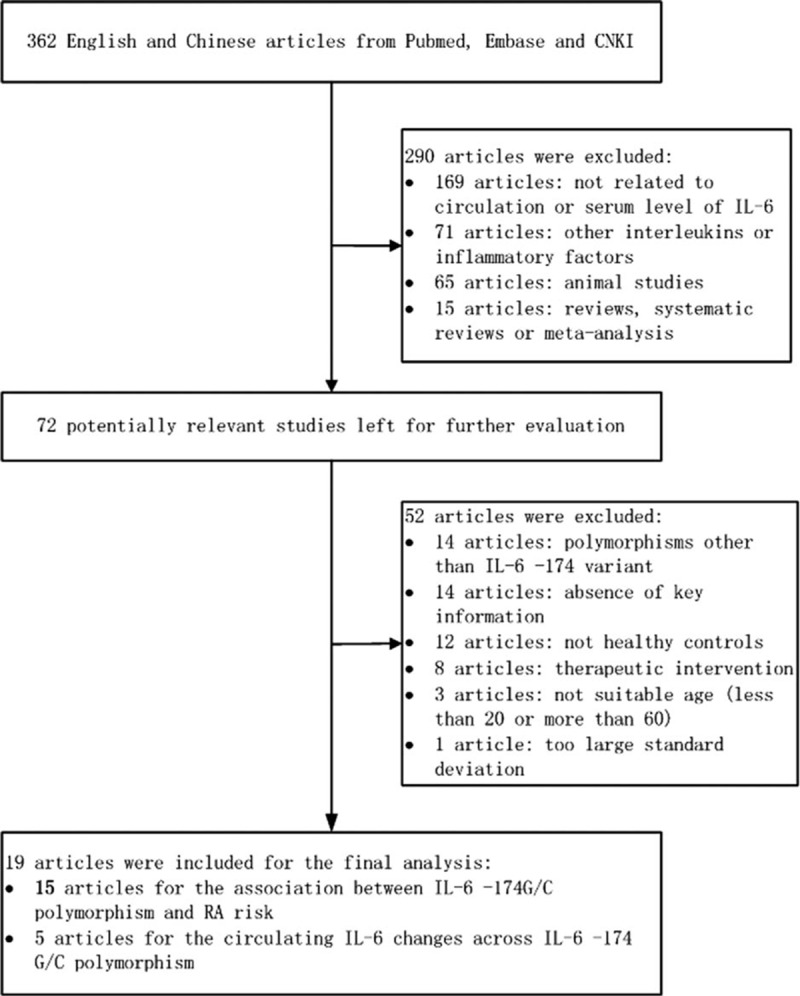
Flow diagram of the search strategy and study selection.

The flow chart of the selection process is shown in Fig. 1. Altogether, 362 potentially relevant articles were identified after the initial research. Finally, 20 of them written in English or Chinese were deemed eligible according to the inclusion criteria. Fifteen articles examined the association of the *IL-6 -174*G/C variant with RA risk,^[24–38]^ and 5 articles studied circulating IL-6 changes across -174G/C genotypes.^[19,35,39–41]^ The publication years ranged from 2000 to 2014, and the total sample size ranged from 32^[40]^ to 1550.^[27]^

### 3.2 Baseline characteristics

Baseline characteristics of studies for the association of *IL-6* promoter gene -174G/C variants with RA risk and circulating IL-6 level are presented in Tables 1 and 2, respectively.

**Table 1:**
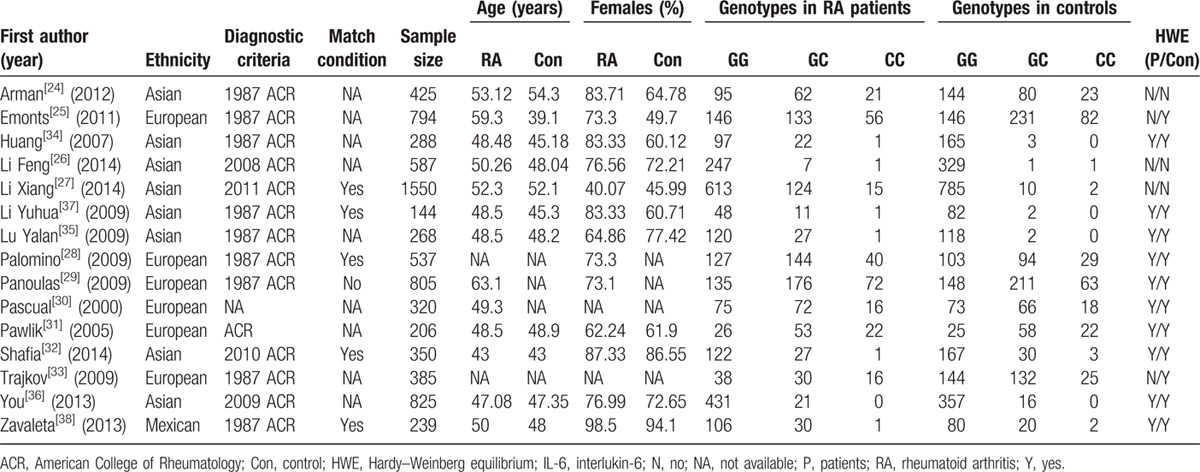
Basic characteristics of eligible studies included in this meta-analysis (IL-6 -174G/C variant and rheumatoid arthritis risk).

**Table 2:**
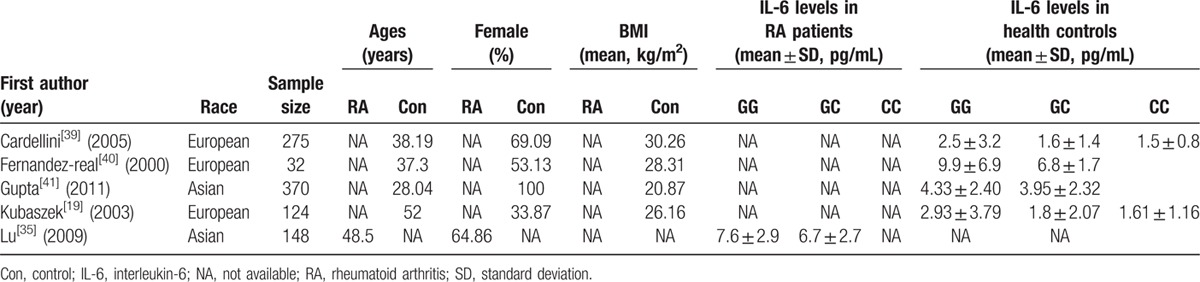
Basic characteristics of eligible studies included in this meta-analysis (*IL-6 -174G/C* variant and circulating IL-6 level).

For the *IL-6* gene variant-RA risk association articles, there were 3629 RA patients and 4092 healthy controls. Seven study were conducted in Asians,^[26,27,32,34–37]^ 7 in Europeans,^[24,25,28–31,33]^ and 1 in Mexicans.^[38]^ The average ages of RA patients and healthy controls were 50.88 and 47.22 years, respectively, with a statistical difference between the 2 groups (*P* < 0.05). No significant difference was found for the percentage of females between RA patients and controls (75.12% vs. 67.83%, *P* > 0.05). Fourteen studies used the Classification Criteria of the American College of Rheumatology as diagnostic criteria for RA,^[24–29,31–38]^ and 1 study did not describe the diagnostic criteria.^[30]^ Age and gender were reported to be matched in 5 studies^[27,28,32,37,38]^ and unmatched in 1 study^[29]^ between RA patients and controls. No related information was provided in the other 9 studies.^[24–26,30,31,33–36]^ Among the 15 studies on the association of the *IL-6* -174G/C variants with RA risk, 10 study groups were in HWE,^[28–32,34–38]^ and the other 5 study groups were found to have a significant departure from HWE.^[24–27,33]^

For the 5 studies related to change in the circulating IL-6 level across *IL-6*-174G/C variants, 3 groups involved Europeans^[19,39,40]^ and the other 2 groups included Asians.^[35,41]^ Four groups provided circulating IL-6 in healthy controls,^[19,39–41]^ and 1 group in RA patients.^[35]^

### 3.3 Association of IL-6 -174G/C variants with RA risk

**Figure 2:**
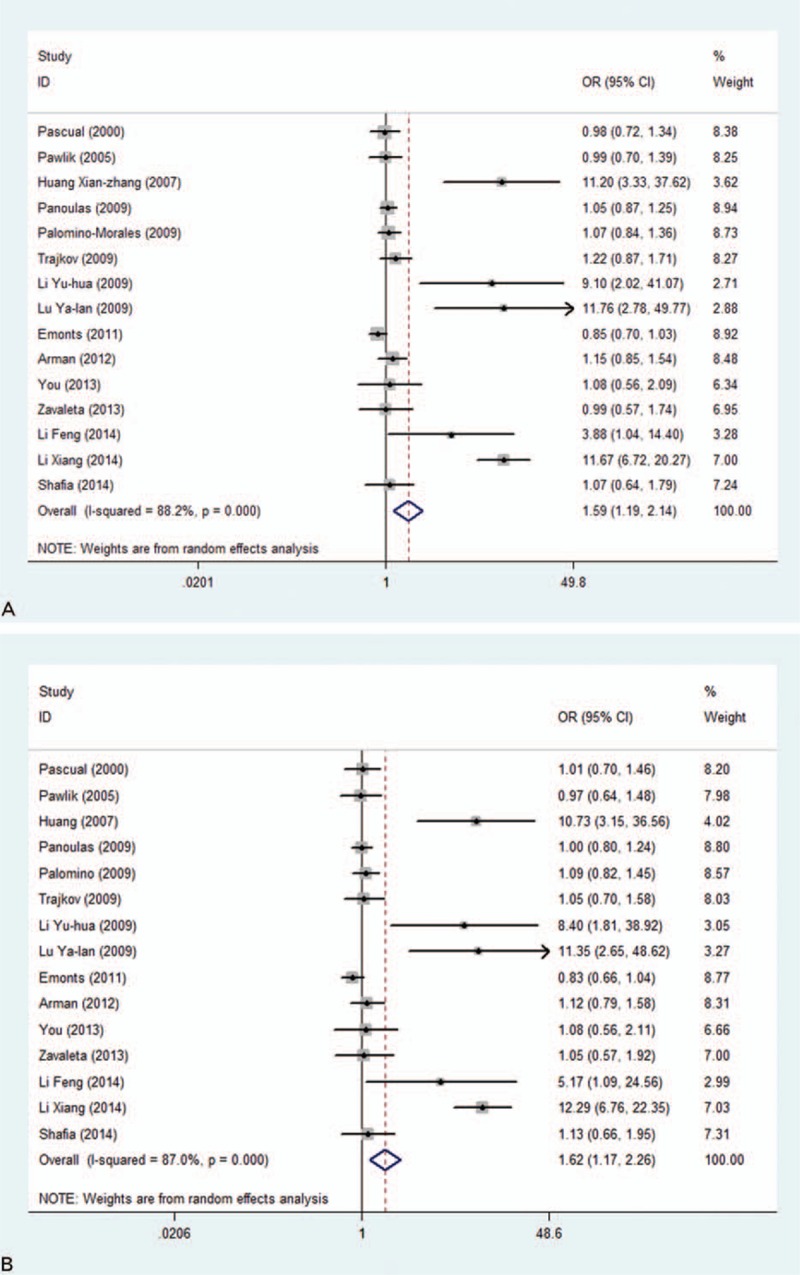
Overall comparisons of interleukin-6 (*IL-6*) *-174* gene C versus G (A) and genotype GC+CC versus GG (B) in association with RA risk.

**Figure 3:**
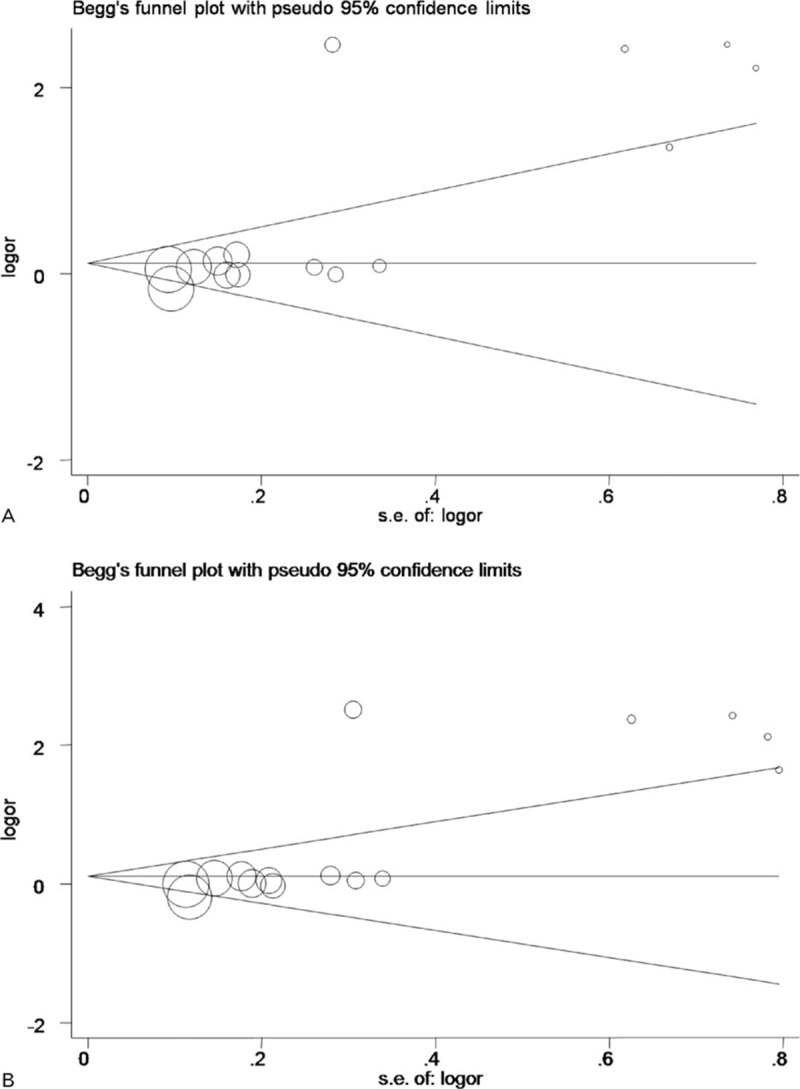
Begg funnel plot analysis to detect publication bias for the comparisons of interleukin-6 (*IL-6*) *-174* gene C versus G (A) and genotype GC+CC versus GG (B).

Taken together, the analyses presented significant association of the -174C allele with RA risk, under allelic (OR = 1.59; 95% CI: 1.19–2.14, *P* < 0.05), and dominant model (OR = 1.625; 95% CI: 1.17–2.26, *P* *<* *0.05*), with high heterogeneity (*I*^2^ = 88.2% and 85%, respectively) (Fig. 2). High publication bias for both genetic models were reflected by the Begg funnel plots (Fig. 3) and Egger tests (*P* = 0.006 for allelic model and 0.005 for dominant model). After restricting study groups with HWE, there was no material change in effect estimates.

In an attempt to exploit the potential sources of heterogeneity, subgroup analysis was conducted according to ethnicity, region (Europe, Eastern China, Western China, Kashmir, and Mexico), sample size (large: more than 500; small: less than 500), and matching condition, separately. To avoid random results, only subgroups with 3 or more groups were considered in this meta-analysis. By ethnicity, the *IL-6*-174C allelic, homozygous, and dominant models are significantly associated with 4.55-fold (95% CI: 1.62–12.75; *P* = 0.003), 1.84-fold (95% CI: 1.13–2.99; *P* = 0.014), and 4.69-fold (95% CI: 1.68–13.14; *P* = 0.003) increased risk of RA in Asian populations, respectively, with high heterogeneity for C allelic (*I*^2^ = 90.3%), dominant model (*I*^2^ = 89.1%), and low heterogeneity for homozygous model (*I*^2^ = 17.2%). No significance was attained in Europeans under 3 genetic models.

Based on the region of origin, significance was only attained for studies in eastern China, with significantly increased risk of RA in carriers of **-**174C allele (OR = 10.05; 95% CI: 6.86–16.08; *P* = 0.000), homozygous model (OR = 5.09; 95% CI: 1.82–14.26; *P* = 0.002), and dominant model (OR = 10.73; 95% CI: 6.79–16.97; *P* = 0.000). This significance was less likely interpreted by heterogeneity (*I*^2^ = 0.0%, 0.0%, and 0.0%, respectively) and was still maintained for all 3 genetic models after Bonferroni correction (*P* < 0.05/5, 5 equals to the number of subgroup by region). Subgroup analysis by sample size and matching condition did not present any significantly difference from the unity under 3 genetic models (all *P* > 0.05), and no considerable improvement in heterogeneity was observed within these subgroups.

### 3.4 Association of IL-6 -174G/C variants with circulating IL-6 level

**Figure 4:**
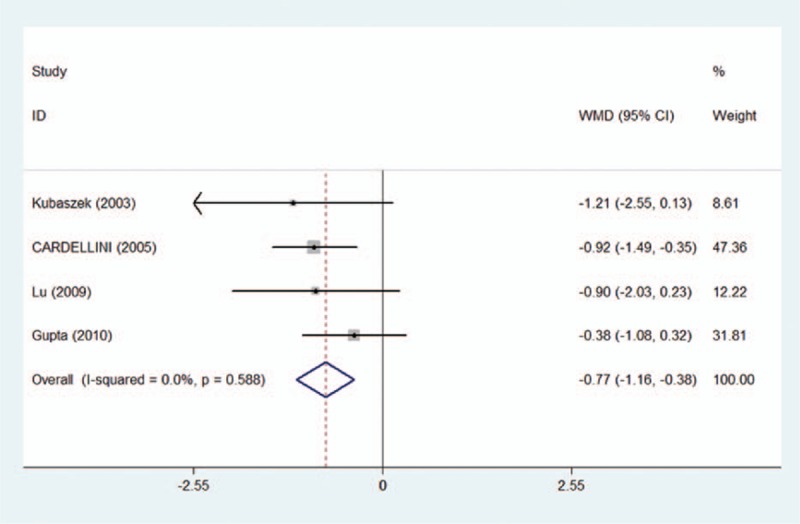
Meta-analysis for the association of the *IL-6 -174*G/C variants and circulating IL-6 level under the dominant model (GC+CC vs GG).

Carriers of the IL-6 -174 GC+CC genotype showed significant reduction in the circulating IL-6 level (WMD = −0.77; 95% CI: −1.16 to −0.38; *P* = 0.000) when compared with carriers of GG genotype (Fig. 4). Conversely, a significantly increased circulating IL-6 level (WMD = 0.64; 95% CI: 0.26–1.03; *P* = 0.001) was observed in carriers of GG+GC genotype compared with carriers of the CC genotype. No significant heterogeneity was found for both comparisons (*I*^2^ = 0.0% and 17.4%, respectively). Pooled analysis was not performed for the comparisons of CC with GG genotype, because of the small number of involved studies (less than 3).

### 3.5 Causal prediction of circulating IL-6 for RA

Under the assumption of Mendelian randomization, 0.1 pg/mL reduction of circulating IL-6 was associated with a 6% (95% CI: 1.04–1.12) and 22% (95% CI: 1.14–1.50) increased risk of RA in overall and Asian populations, respectively. In eastern China, this association became even stronger with a 0.1 pg/mL reduction of IL-6 corresponding to a 36% increased risk of RA (95% CI: 1.23–1.87). Considering that the null hypothesis value of 1 was not included, it is safe to reject that there is no causal relevance between circulating IL-6 and RA risk.

### 3.6 Sensitivity analysis

Sensitivity analysis, which was performed by sequentially omitting each study once at a time and computing the differential estimates of remaining studies, confirmed the pooled effect estimates regarding the associations of *IL-6* gene -174G/C variant with RA risk and circulating IL-6 level in both direction and magnitude.

## 4 Discussion

In the current Mendelian randomization meta-analysis of 20 eligible articles, the *IL-6* 174G/C variant was used as the surrogate marker to evaluate the causal relevance between circulating IL-6 level and RA risk. Our results indicated that long-term genetically reduced IL-6 might be causally associated with a higher risk of RA. To the best of our knowledge, it is the first meta-analysis to evaluate the differences of circulating IL-6 on RA risk to date.

Evidence from observational case–control studies has demonstrated a positive association between circulating IL-6 and RA.^[3–7]^ However, it is not clear whether this association is causal, because of the involvement of many confounding factors, such as BMI, age, and genetic backgrounds, as well as the complex biological effects of IL-6, which are in tractable in classic epidemiology. Mendelian randomization, which is deemed as more similar to randomized clinical trials due to the Mendel 2nd law, has been introduced as a viable technique to overcome drawbacks of observational epidemiological studies and obtain robust causal estimates.^[10]^ Therefore, the Mendelian approach was employed to evaluate the causal relevance of IL-6 with RA risk, with focus on the *IL-6* -174G/C variant.

In the current meta-analysis, the associations of *IL-6* gene variant with RA presented heterogeneity between Europeans and Asians, and between Eastern China and other regions. This divergence might be most likely explained by the different genetic background or linkage disequilibrium. For instance, the -174C allele and its related genotype were exceedingly lower in Asians and Eastern China than in Europeans and other regions, respectively. Actually, it is not rare that 1 gene variant plays a different role in RA risk across different populations and regions,^[42–44]^ which is the principle limitation of this Mendelian meta-analysis. Therefore, if there is linkage disequilibrium between -174G/C and other flanking variants within or near *IL-6* gene in 1 population and/or region but not in another, our findings will be influenced. This would lead to divergent results across different populations or regions, which is difficult to exclude completely. In addition, significant heterogeneity, which was observed in Asian populations, was not found in population from Eastern China, indicating region to be one of the major source of heterogeneity. Thus, these findings suggest the necessity of establishing the population and region-specific database of potential genes and variants for RA risk.

The lower circulating IL-6-associated GC+CC genotype is found to be a risk factor for RA in Asians, rather than a protective agent. There is a negative causal relevance of circulating IL-6 levels with RA risk in both Asians and overall populations, which is likely contradictory to previous observational studies.^[5–7]^ Currently there is no clear explanation for this discrepancy. However, the low statistical power caused by exceedingly low frequency of C allele in Asians could be one of the explanations, indicating that studies with large populations, especially Asians, is necessary to obtain robust conclusions. Besides, participants in 4 of total 5 studies analyzing the association of IL-6 -174 variants with circulating IL-6 level were healthy controls, suggesting that the negative causality is more reflective of condition in normal controls.^[19,39–41]^ Considering the functional pleiotropy of IL-6 (both pro- and antiinflammatory)^[9]^ and the absence of long-term cohort epidemiological study, we cannot deny the potential protective role of IL-6 for RA risk in health controls. In addition, recent findings demonstrated that IL-6 presented both anti- and proinflammatory functions in “normal” and “stimulated” peripheral blood mononuclear cells, respectively, suggesting different roles of circulating IL-6 in different stages of disease.^[45]^ Therefore, it is possible that serum IL-6 can act as both an inflammatory cytokine (high levels of which was observed in RA patients) and an antiinflammatory factor (genetically induced decrease of which could cause increased risk of RA in normal populations).

Some limitations in this meta-analysis should be acknowledged. First, the research scope of this meta-analysis is restricted to published articles written in English and Chinese, leading to the potential selective publication bias. Second, the data of circulating IL-6 level across -174G/C variants were mostly from healthy controls, which cannot reflect the situation in RA patients, indicating more involvement of studies in RA patients. Third, circulating IL-6 level was measured only once in almost all involved studies, which cannot reflect its long-term role in the development of RA. Fourth, only IL-6 -174G/C variant was selected in the current meta-analysis. Whether the predicted causal relevance calculated with other candidate genes or polymorphisms will be consistent with the findings of the current study will be challenging. Fifth, the potential pleiotropic effect of IL-6 -174G/C variants cannot be excluded in this meta-analysis, which may lead to seriously biased causation between circulating IL-6 level and RA risk. Therefore, the conclusion of this meta-analysis should be treated cautiously, until large, well-designed prospective studies confirm our findings.

Generally speaking, this Mendelian randomized meta-analysis indicates that the reduced circulating IL-6 level might be a causal risk factor for RA in both Asians and overall populations, which provided further understanding of the association between IL-6 and RA. However, considering the limitations mentioned above, more investigations with optimized background will be necessary to confirm our findings.
